# Predictors of lymphovascular invasion in estrogen receptor positive/Her-2 negative breast cancer patients treated with neoadjuvant chemotherapy

**DOI:** 10.55730/1300-0144.5414

**Published:** 2022-03-19

**Authors:** Eyyüp ÇAVDAR, Yakup İRİAĞAÇ

**Affiliations:** Department of Medical Oncology, Faculty of Medicine, Tekirdağ Namık Kemal University, Tekirdağ, Turkey

**Keywords:** Breast cancer, lymphovascular invasion, predictive, chemotherapy

## Abstract

**Background/aim:**

Lymphovascular invasion (LVI) is considered a high-risk factor for recurrence in early-stage breast cancer, hence examination of LVI in pathological samples is an absolute recommendation. We aim to investigate predictive factors of LVI in pre-neoadjuvant chemotherapy (NAC) patients with estrogen receptor positive (ER+) and human epidermal growth factor receptor 2 negative (HER2−) molecular subtypes of breast cancer.

**Materials and methods:**

One hundred and thirty-four patients treated with NAC were included in this study who were ER+/HER2−. The clinical characteristics of the patients, the data obtained from the core needle biopsy before NAC and the LVI status in the pathology that examined after breast surgery were collected. Univariate and multivariate analysis were performed using the logistic regression model.

**Results:**

An examination of the association between LVI and clinical-pathological patient characteristics showed that advanced age (>40 years old) (p = 0.021), ductal histology (p = 0.039), and presence of axillary lymph node metastasis (p = 0.005) were predictors of LVI. Independent predictors of LVI in a multivariate logistic model included advanced age (p = 0.037), and the presence of axillary lymph node metastasis prior to NAC (p = 0.006). The median RFS (Recurrence-free survival) time was 22.8 months for all patients. RFS was shorter in patients with LVI (log-rank p = 0.037).

**Conclusion:**

Independent predictors of LVI are advanced age and lymph node positivity at the time of diagnosis. Our study is the first study that evaluates pre-NAC predictive factors of LVI in ER+/HER2− breast cancer patients treated with NAC.

## 1. Introduction

Neoadjuvant chemotherapy (NAC) is one of the recommended treatments in patients with locally advanced breast cancer and inflammatory breast cancer. It has been well-established that the chance of breast-conserving surgery (BCS) and pathological complete response is increased following shrinkage of tumor with neoadjuvant therapy [1,2]. Responses given to neoadjuvant therapy vary depending on the phenotype therefore, the prognosis of breast cancer also varies depending on the response. Nowadays, molecular subtyping has become more important, patient subgroups with the poorest response to chemotherapy are known to Luminal A like tumors (ER +, HER2−) [3,4]. NAC may provide earlier identification of chemo-sensitivity of ER+, HER2− and other molecular subtypes of breast cancer [5].

Patient age, T and N status at staging, molecular subgroup, histological grade, hormone receptor status, lymphovascular invasion (LVI), and pathological complete response (pCR) following NAC are considered as well-established prognostic factors in breast cancer [6–10]. LVI has been identified as a prognostic factor in operated breast cancer patients regardless of axillary involvement, although the mechanisms of such association have not been fully elucidated [9,11]. Therefore, in major oncology guidelines, LVI is considered a high-risk factor for recurrence in early-stage breast cancer, hence examination of LVI in pathological samples is an absolute recommendation [1,12]. Despite this, studies examining LVI following NAC are scarce in number, and to the best of our knowledge, no previous studies have looked at the relationship between LVI and pre-NAC clinico-pathological characteristics of the patients. Previous studies detected LVI by examining the samples that were obtained from surgery. Additionally, analysis of histopathological factors associated with LVI has been performed with the same specimen. This analyzing method could avoid the detection of the true predictive factors of LVI since presurgical NAC treatment could affect the Ki-67 index and receptor expression. The objective of this study was to explore pretreatment factors predicting LVI status in patients undergoing NAC due to nonmetastatic luminal breast cancer who were ER +/HER2−, which are known to be associated with poorer response to chemotherapy as compared to other molecular subtypes.

## 2. Materials and methods Patients

Breast cancer patients undergoing NAC prior to surgery at Tekirdağ Namık Kemal University Hospital between 1 January 2016 and December 2021 were retrospectively examined upon receiving approval from the relevant ethics committee. Magnetic resonance imaging (MRI), abdominal ultrasound and chest X-ray were utilized to determine distant metastases, contralateral breast lesions, and disease stage. Positron emission tomography-computed tomography (PET-CT) was taken in patients with suspected metastasis. Exclusion criteria included subjects under 18 years of age, male subjects, HER2 receptor positivity and/or estrogen receptor (ER) negativity, use of different neoadjuvant treatments, and presence of findings suggesting metastasis.

### 2.1. Treatment

Indications for neoadjuvant therapy were as follows: clinically node positive, pathologically confirmed nodal metastases on lymph node biopsy, and primary tumor size ≥5 cm. All cases were discussed at the Institutional Multidisciplinary Tumor Board. Patients receiving 4 cycles of cyclophosphamide (600 mg/m^2^) + epirubicin (90 mg/m^2^) followed by either docetaxel (75 mg/m^2^) every 3 weeks for 4 cycles or weekly paclitaxel (80 mg/m^2^) for 12 cycles were included. All patients had undergone surgery following completion of NAC. All patients were treated with hormone therapy after surgery and adjuvant radiotherapy was given to eligible patients in collaboration with a radiation oncologist.

### 2.2. Pathology

Histological subtyping, SMA expression, CK7 expression, P63 expression, E-cadherin expression levels, Ki-67 and grading were based on biopsy samples obtained prior to NAC. Based on the American Society of Clinical Oncology/College of American Pathologists guidelines, patients whose ER and PgR (progesterone receptor) levels were higher than 1% were considered positive [13]. Patients were considered HER2 positive and thus excluded from the study, if HER2 immunohistochemical (IHC) analyses showed a score of +3, or a score of +2 with positive fluorescence in situ hybridization (FISH) analysis. ER expression was divided into two groups according to ASCO/CAP guidelines: ER > 10% (non-low expression) and ER ≤ 10% (low expression) [14]. Ki-67 expression was divided into two groups: Ki-67 ≥ 14% (high expression) and Ki-67 < 14% (low expression). This cut-off was determined according to Gallen International Expert Consensus [15]. The same cut-off was used for molecular subtyping. Axillary lymph node positivity was verified histopathologically.

### 2.3. Definition of LVI

**Figure 1:**
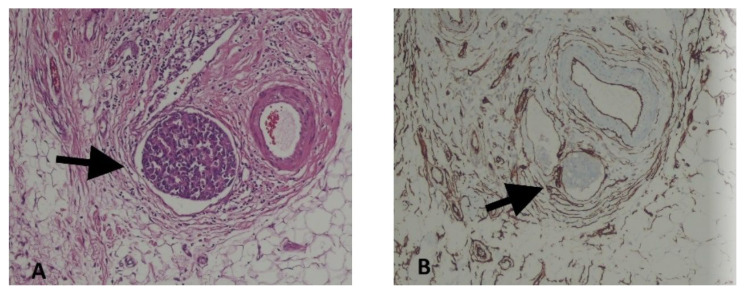
Lymphovascular invasion (black arrow) seen in hematoxylin-eosin (A) and CD34 (B) stained sections of breast tumors from the same case; ×400.

LVI positivity was defined as the presence of tumor cells within an endothelium-lined space (lymphatics or blood vessels) as demonstrated by hematoxylin-eosin staining and IHC on surgical slides after NAC. For inconclusive cases, a specific marker (CD34) was utilized (Figure 1A, 1B).

### 2.4. Statistical analysis

The Fisher exact test and the Mantel–Haenszel chi-square test for trend were used to assess the association between categorical or ordinal variables and the presence of LVI. Univariate and multivariate analyses were performed using the logistic regression model. Odds ratio (OR) was reported with the corresponding 95% confidence intervals (95% CI) and p < 0.05 was considered statistically significant. Statistical analyzes were performed using SPSS Statistic software 24 (SPSS Inc., Chicago, III). To predict LVI, binary logistic regression using the “Forward:LR” method was used for multivariate analyses. Times of recurrence-free survival (RFS) was calculated according to the Kaplan-Meier method from the date of surgery to the occurrence of local recurrence or distant metastasis.

### 2.5. Ethical approval

The study was approved by the Tekirdağ Namık Kemal University ethics committee in accordance with the Helsinki declaration.

## 3. Results

A total of 134 patients were included in the study. The median age was 50 years (range: 38–79 years). Sixty-three (47.0%) patients had LVI positivity. In the classification made according to molecular status, 25 (18.7%) patients were Luminal A and 109 (81.3%) patients were Luminal B(Her2−). Table 1 shows the association between LVI status and clinicopathological patient characteristics.

A regression analysis was performed to assess the association of clinicopathological data with LVI. Age over 40 years, ductal tumor histology, and presence of axillary lymph node metastasis prior to neoadjuvant chemotherapy were identified as predictors of LVI in univariate analysis (p = 0.021, p = 0.039, and p = 0.005, respectively). When a multivariate logistic model was applied, independent predictors of LVI were identified as advanced age (OR 2.69; 95% CI, 1.06–6.83; p = 0.037), and presence of axillary lymph node metastasis prior to NAC (OR 8.37; 95% CI, 1.82–38.52; p = 0.006). The results are shown in Table 2.

**Figure 2:**
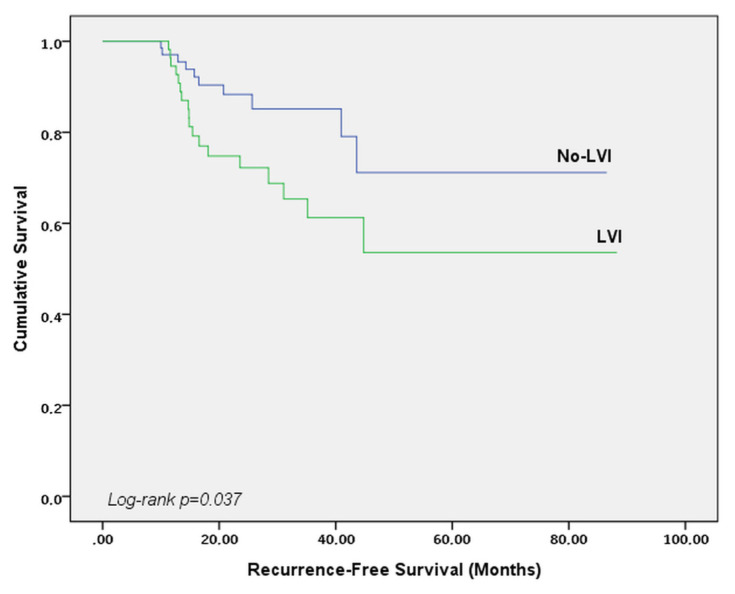
Kaplan Meier survival curve by LVI status for recurrence-free survival (RFS).

The median follow-up period of the patients after breast surgery was 43.7 months. Twenty-eight (20.9%) patients had recurrence (local or distant metastasis) during the follow-up period. The median RFS (mRFS) in all patients was 22.8 months (95% CI: 24.2–30.4). mRFS was 19.5 months in LVI positive patients, 23.3 months in LVI negative patients (log-rank p = 0.037) (Figure 2).

## 4. Discussion

The response rate to NAC in ER +/HER2− luminal breast cancer subtypes is only one third of that in hormone negative molecular subtypes, and therefore these patients are generally considered chemotherapy-resistant [16–18]. In ER +/HER2− tumors showing inadequate response to chemotherapy, the survival benefit even in the presence of pathological complete response (pCR) is controversial [16,19]. In recent years, there has been an increase in the number of studies demonstrating a prognostic role for LVI in ER +/HER2− groups [9,20–24]. LVI is considered to represent an important step in tumor progression and metastasis [11,25].

In the literature, there are limited studies on the factors that predict LVI in breast cancer. Previous studies detected LVI by examining the samples that were obtained from surgery. Additionally, analysis of histopathological factors that are associated with LVI has been performed with the same specimen. Our study was designed differently and analyzed potential predictive histopathological factors of LVI based on data from a reexamination of patients’ pre-NAC biopsies. According to our results advanced age, ductal histology, and presence of lymph node metastasis were predictors of LVI. Among all the predictors, advanced age and lymph node metastasis emerged as independent predictors.

Zhao et al. evaluated the patients that were not treated with NAC before breast surgery and found that younger patients had a higher frequency of LVI than older patients [26]. However, Rakha et al. reported that LVI was frequently seen in advanced ages [11]. Ryu et al. included the patients that were treated with NAC before surgery and did not find relation between age and LVI [27]. There is no consensus on this issue in previous studies. In our study, we found that advanced age is an independent predictive factor of LVI. LVI was 2.69 times more common in patients over 40 years of age than patients under 40 years of age.

Axillary lymph node metastasis was reported to be associated with LVI [27–31]. However, previous studies designed differently and examined the relationship between pathological N status and LVI. In our study, pre-NAC axillary positivity was an independent predictor of LVI. In studies of Muhammed et al. and David et al., the presence of LVI was reported to be a significant predictor for histological invasion in regional lymph nodes [32,33]. In Ran et al.’s study as well as a meta-analysis by Zhang et al. involving 2920 patients, LVI and axillary lymph node metastases were found to be interrelated sequential steps that ultimately result in metastasis. It is unknown whether LVI is a reason for lymph node metastasis or a result of lymph node metastasis. However, it can be suggested that axillary lymph node metastasis and LVI status, which are correlated and considered to be developing via similar mechanisms, could predict each other as a result of the involvement of similar pathways. Axillary lymph node metastasis is an important component of the TNM staging system and is considered a prognostic marker for breast cancer and is strongly associated with LVI. This association between axillary lymph node metastasis and LVI supports the clinical significance of LVI in ER+/HER2 negative breast cancer patients [34–36].

In our study, we did not identify an independent association with menopause, histological type, hormone receptor levels, Ki-67, tumor size, and disease grade, which were reported to be associated with LVI in previous studies [28,29,32,37–42]. The lack of such association may be related to the fact that previous studies generally included patients with all molecular subtypes of breast cancer, and used different cut-off values for Ki-67 and hormone receptors. Alternatively, it may be related to the fact that our study utilized a different design with the analysis of pre-NAC factors which might have been altered due to NAC and lost their significance. Accordingly, alterations in Ki-67, PgR, and ER have been shown to occur in association with chemotherapy [43–46].

One limitation of our study was the use of IHC for molecular subtyping, which allows genotype-based breast cancer subtyping only, and this might lead to a certain degree of misclassification of tumors. In our study, patients with rare subtypes of breast cancer were limited in number, and more recent analytic techniques involving genomic testing were not utilized [47,48]. Additionally, core needle biopsy has a limited representation of all breast cancer tissue due to the small tissue volume. As far as we know, our study is the first that analyzes pre-NAC variables. Furthermore, CK-7, SMA, and p68 expression in breast cancer patients were analyzed for the first time in terms of LVI. Our findings are also significant due to the examination of pre-NAC variables specifically in a subgroup of ER +/HER2− subjects. Further prospective and more comprehensive studies with larger sample sizes or meta-analyses are required to reach firmer conclusions regarding LVI-related factors.

In conclusion, this study showed that advanced age and pre-NAC N status are independent predictive factors of LVI. We need further studies to examine the predictive factors of LVI, which is considered to be a prognostic marker for survival, particularly in ER+/HER2− molecular subtype of breast cancer with poor response to chemotherapy. Identification of predictors may pave the way for further studies in terms of disease monitoring and potential therapeutic target therapy.

## Figures and Tables

**Table 1:** Patient distribution according to clinicopathological characteristics and LVI status.

Clinicopathological characteristics	Total (n, %)	LVI (−) n = 71	LVI (+) n = 63
**Age**			
<40 (Young adult)	29 (21.7%)	21 (72.4%)	8 (27.6%)
≥40	105 (78.3%)	50 (47.6%)	55 (52.4%)
**Molecular subtype**			
Luminal A	25 (18.7%)	9 (36.0%)	16 (64.0%)
Luminal B(HER2−)	109 (81.3%)	63 (57.8%)	46 (42.2%)
**Histologic type**			
Ductal	109 (81.3%)	53 (48.6)	56 (51.4%)
Others	25 (18.7%)	18 (72.0%)	7 (28.0%)
**ER status**			
<10%	10 (7.5%)	7 (70.0%)	3 (30.0%)
≥10%	124 (92.5%)	64 (51.6%)	60 (48.4%)
**PgR status**			
Negative	19 (14.2%)	14 (73.7%)	5 (26.3%)
Positive	115 (85.8%)	57 (49.6%)	58 (50.4%)
**Ki-67**			
<14%	33 (24.6%)	14 (42.4%)	19 (57.6%)
≥14%	101 (75.4%)	57 (56.4%)	44 (43.6%)
**Menopausal status**			
Premenopausal	61 (45.5%)	37 (60.9%)	24 (39.1%)
Postmenopausal	73 (54.5%)	34 (46.6%)	39 (53.4%)
**Grade**			
Grade 1	14 (10.4%)	8 (57.1%)	6 (42.9%)
Grade 2	81 (60.5%)	41 (50.6%)	40 (49.4%)
Grade3	39 (29.1%)	22 (56.4%)	17 (43.6%)
**Pre-NAC tumor size**			
<2cm	28 (20.9%)	14 (50.0%)	14 (50.0%)
≥2cm	106 (79.1%)	57 (53.8%)	49 (46.2%)
**Pre-NAC lymph node status**			
Negative	18 (13.4%)	16 (88.9%)	2 (11.1%)
Positive	116 (86.6%)	55 (47.4%)	61 (52.6%)
**CK-7 expression**			
Negative	20 (15.0%)	11 (55.0%)	9 (45.0%)
Positive	114 (85.0)	60 (52.6%)	54 (47.4%)
**p63 expression**			
Negative	111 (82.8%)	55 (49.5%)	56 (50.5%)
Positive	23 (17.2%)	16 (69.6%)	7 (30.4%)
**E-cadherin expression**			
Negative	24 (17.9%)	16 (66.7%)	8 (33.3%)
Positive	110 (82.1%)	55 (50.0%)	55 (50.0%)
**SMA expression**			
Negative	107 (79.9%)	53 (49.5%)	54 (50.5%)
Positive	27 (20.1%)	18 (66.7%)	9 (33.3%)

LVI, Lymphovascular invasion; ER, Estrogen receptor; PgR, Progesterone receptor; NAC, Neoadjuvant chemotherapy; HER-2, Human epidermal growth factor receptor 2; CK-7, Cytokeratin 7; SMA, Smooth muscle actin.

**Table 2:** Univariate and multivariate analyses of factors for LVI in ER-positive/HER2-negative patients with neoadjuvant chemotherapy.

		Univariate analysis		Multivariate analysis	
Variable	Category	OR (95% CI)	p	OR (95% CI)	p*^f^*
Age	<40/≥40	2.89(1.17–7.10)	**0.021**	2.69(1.06–6.83)	**0.037**
Molecular subtype	LuminalA/B(HER2−)	0.43(0.17–1.05)	0.064		
Histologic type	Ductal/others	0.37(0.14–0.95)	**0.039**		
ER status	<10%/≥10%	2.19(0.54–8.85)	0.272		
PgR status	Negative/positive	2.85(0.96–8.43)	0.058		
Ki-67	<14%/≥14%	0.57(0.26–1.26)	0.164		
Menopausal status	Pre/post	1.77(0.89–3.52)	0.105		
Grade	1/2/3	0.94(0.53–1.65)	0.828		
Pre-NAC T size	<2cm/≥2cm	0.86(0.37–1.98)	0.722		
Pre-NAC N status	Negative/positive	8.87(1.95–40.34)	**0.005**	8.37(1.82–38.52)	**0.006**
Ck-7 expression	Negative/positive	1.10(0.42–2.86)	0.845		
p63 expression	Negative/positive	0.43(0.16–1.13)	0.086		
E-cadherin expression	Negative/positive	2.00(0.79–5.06)	0.143		
SMA expression	Negative/positive	0.49(0.20–1.19)	0.115		

s Significant values are indicated in bold. P^f^: Forward: LR method LVI, lymphovascular invasion; HER-2, Human epidermal growth factor receptor 2; ER, Estrogen receptor; PgR, Progesterone receptor, NAC, Neoadjuvant chemotherapy; CK-7, Cytokeratin 7; SMA, Smooth muscle actin.
